# Advancing regulatory science, advancing regulatory practice

**DOI:** 10.1002/pds.4181

**Published:** 2017-02-21

**Authors:** Xavier Kurz

**Affiliations:** ^1^ European Medicines AgencyDepartment Pharmacovigilance and EpidemiologyLondonUK

In an Editorial of *Science* in 2011, Margaret A. Hamburg, the then Commissioner of the US Food and Drug Administration highlighted that a strong field of regulatory science is required to develop new tools, standards and approaches that efficiently and consistently assess the safety, efficacy and performance of products, and that this field has long been underappreciated and underfunded.^1^ During the last decade, several international initiatives were taken to translate results of scientific research into everyday regulatory practice. The US Food and Drug Administration launched the Advancing Regulatory Science Initiative to transform the way medical products are developed, evaluated and manufactured.^2^ The Innovative Medicines Initiative (IMI) was launched in Europe as a public–private partnership aiming to improve health by speeding up the development of, and patient access to, innovative medicines, particularly in areas where there is an unmet medical or social need.^3^, ^4^ Improving and strengthening the monitoring of the benefit–risk of medicines marketed in the European Union (EU) was one of the first topics adopted by IMI in 2008. Its main goal was to enable a more rapid detection and confirmation of new adverse drug reactions (ADRs) under ‘real world’ conditions and to develop new scientifically based and tested tools for the benefit–risk assessment of marketed drugs. The European Medicines Agency (EMA) considered it should play a role in this project and established the Pharmacoepidemiological Research on Outcomes of Therapeutics by a European ConsorTium (PROTECT) group to which the project was eventually awarded in association with a consortium of pharmaceutical companies coordinated by GlaxoSmithKline. Pharmacoepidemiological Research on Outcomes of Therapeutics by a European ConsorTium was conducted from 1 September 2009 to 28 February 2015 with 33 other organisations and pharmaceutical companies.^5^

In terms of research *outputs*, PROTECT was in no doubt successful, with more than 75 original publications in peer‐reviewed journals and 100 presentations in various conferences and meetings.^6^ Projects from PROTECT were also the subject of 14 doctoral theses and three master theses carried out in universities across the EU. But in regulatory science, the number of reports, presentations, publications or databases should not be an ultimate marker of success: the project should generate *outcomes* in the form of strengthened regulatory systems that ensure patient safety, enhance public health and stimulate innovation.^7^ This article briefly reviews four examples of PROTECT results and discusses the different impact on regulatory practice and public health the results have already had or may have in the future.

### Good signal detection practices

Pharmacoepidemiological Research on Outcomes of Therapeutics by a European ConsorTium conducted original research into the performance of different signal detection methods in order to support recommendations that can be converted into meaningful and implementable applications.^8^ It led to significant improvements of the performance and efficiency of signal detection from spontaneous reports based on findings that, for a given threshold, the choice of a disproportionality statistic can be primarily based on ease of implementation, interpretation and optimisation of resources, and the choice of this threshold can be driven by the balance between earliness, number of detected signals and amount of resources needed. Subgrouping of spontaneous reports by age and country of origin also achieves a higher level of performance than stratification. In addition, although electronic health record databases were found potentially useful for signal detection, their use may be constrained by limitations in size and scope (which drugs and diagnoses they capture) and the need for clinical, pharmacological and epidemiological reviews of the identified medical events. As part of this work on signal detection, PROTECT created the ADR Database, a public downloadable Excel file of all ADRs listed in the Summary of Product Characteristics of medicinal products authorised in the EU according to the centralised procedure, converted into MedDRA preferred or low‐level terms.^9^ It also includes available information on gender, causality, frequency and class warning. Based on the work done by PROTECT, major changes were introduced in 2016 in Europe in the format of the electronic Reaction Monitoring Report (eRMR). The eRMR is the principle tool used for signal detection in EudraVigilance, the EU database of reports of suspected ADRs,^10^ and is produced on a monthly or bimonthly basis by the EMA for more than 1500 substances authorised in the EU to facilitate the surveillance, detection, evaluation and documentation of suspected ADRs reported in EudraVigilance. Changes in the eRMR concerned the choice of statistical measure and threshold values, provision of detailed statistics by age groups and automated inclusion of listedness information for each drug–ADR combination. Preliminary results of these changes have shown an increased performance of the signal detection process with less false positives and more validated signals. In addition, the PROTECT recommendations are the backbone to new methodological guidance included in revised EU good pharmacovigilance practices (GVP) on signal management (applicable to pharmaceutical companies, national competent authorities and the EMA) and the ENCePP Guide on methodological standards for pharmacoepidemiology.^11^, ^12^ The ADR Database is a major resource used for the translation of lay terms into the MedDRA classification and to evaluate masking effects of known ADRs on the performance of signal detection.

### Methods for multi‐database pharmacoepidemiological studies

The second example concerns methods for pharmacoepidemiological studies, of which PROTECT aimed to significantly improve the design, conduct and analysis, especially in the context of multi‐centre, multi‐database studies. Methodological issues examined included the consistency of findings across study designs and databases, outcome definition, exposure definition, control of confounding and choice of study population. In a special issue of *Pharmacoepidemiology and Drug Safety*,^13^ PROTECT presented key results and further discussed the implications of common study protocols for scientific and operational practice and strategies in choosing between multiple study designs. Recommendations were introduced into revised GVP on post‐authorisation safety studies and the ENCePP Guide. A detailed structured downloadable inventory of drug consumptions databases in 28 European countries was established to facilitate identification of reliable and validated aggregated data sources on drug exposure and estimation of population attributable risks of ADRs.^14^ Discrepant and poor quality observational studies are factors that may delay regulatory decision‐making on drug safety, and it is expected that PROTECT recommendations will contribute to improvements in the overall quality and consistency of studies, increase the confidence in results of observational studies and speed up the conduct of multi‐database studies based on a common‐protocol study approach. Any effect on the quality of regulatory decisions will however be extremely difficult to measure due to the large number of intervening factors.

### Benefit–risk assessment of medicines

Work done on quantitative methods for benefit–risk assessment of medicines represents a third example. From a large number of methodologies for benefit–risk assessment reviewed, classified and appraised, 13 were recommended for future use and tested in eight case studies.^15^, ^16^, ^17^ As there was a lack of consensus on which visual representations were most suitable to display benefit–risk profiles, PROTECT also reviewed, described and illustrated 16 ways in which benefits and risk may be communicated to different target groups in different situations with an evaluation of their strengths and weaknesses.^18^, ^19^ A website was created to present training material on all methods and case studies, accompanied by a specific section providing a guide for patients and members of the public who are new to benefit–risk assessment of medicines.^20^ Through this comprehensive review and evaluation of methods and visualisation techniques on benefits and risks, PROTECT paved the way to further research on methods applied to regulatory decision‐making, developed a framework for benefit–risk assessment, helped to understand the use of patient preferences for decision‐making and supported communication on benefits and risks. In 2007, the EMA's Committee for Human Medicinal Products stated that quantitative benefit–risk assessment is not expected to replace qualitative evaluation and that expert judgement is expected to remain the cornerstone of benefit–risk evaluation for the authorisation of medicinal products.^21^ The implementation of methods described in PROTECT was included in the Benefit‐Risk Methodology Project launched by the Committee for Medicinal Products for Human Use to develop and test tools and processes for balancing multiple benefits and risks as an aid to informed regulatory decisions.^22^, ^23^ Pharmacoepidemiological Research on Outcomes of Therapeutics by a European ConsorTium benefit–risk outputs are widely used in other research projects. For example, research proposals launched by IMI specified that projects with topics as diverse as patient perspective elicitation on benefits and risks of medicinal products and vaccine benefit–risk monitoring should strongly build on the PROTECT outputs. Therefore, although results on benefit–risk assessment did not have an immediate on regulatory practice, they will have a significant influence on the appropriate use of benefit–risk assessment methods in the longer term.

### Data collection through the Internet

As a fourth example, PROTECT piloted a study designed to explore use of the Internet or a telephonic interactive voice response system to assess whether information collected through these channels was complete and accurate enough to be used for pharmacovigilance. This research found a low acceptability of interactive voice response system but showed the added value of the Internet for learning about prescription and non‐prescription medication use and for collecting data regularly during pregnancy, and in some cases these data are more complete than those from prescription registers and electronic health records.^24^ These results are important for studies on medicines in target populations that are difficult to recruit and retain using conventional methods (e.g. pregnant women, adolescents, people in full time work) in a very quickly changing environment where patients are actively sharing information. Given the rapid progress of communication technologies, they are however not directly implementable into practice beyond their general conclusions.

## Discussion

Private sector participation in PROTECT and regulatory science in general may raise questions on potential conflicts of interest because research results may have direct regulatory implications. In PROTECT, this concern was addressed first by design. The selection of drug–adverse event pairs included in case studies for methodological investigations was well‐known ADRs already listed in product information. A policy was also agreed at the initiation of the project that any arising safety issue would be evaluated through the routine signal management process in place at the EMA and its EU pharmacovigilance committee. No such case occurred, but results of a PROTECT study on calcium channel blockers and the risk of cancer^25^ were included in the body of evidence for the pharmacovigilance committee's review of this issue.^26^ For the benefit–risk assessment workstream, all publications and presentations included the disclaimer that the report neither replaced nor intended to replace or comment on any regulatory decisions made by national regulatory agencies or the EMA. In addition, inclusion of new recommendations in regulatory guidance document follows a structured and rigorous process of review by committees and public consultation.^27^ It is unlikely that the outcome of this process could be unduly influenced by any party involved in the original research.

Examples of regulatory science cited by Margaret A. Hamburg in *Science* included novel biomarkers of drug‐induced toxicity, ‘omics’ tools such as genomics and systems biology that can replace toxicological assays and accelerate the evaluation of drug toxicities during the drug development and promote public health.^1^ These tools are readily implementable soon after completion of their development. Of the PROTECT examples presented above, tools for signal detection can be immediately applied in practice and have had the greatest impact on regulatory processes and performance of pharmacovigilance. Nevertheless, the large number of health determinants will make it difficult to single out a distinct effect on drug safety and patient health. Regulatory science encompasses development of methods, standards and approaches, such as methodological improvements in the conduct of drug safety studies, as well as evaluation of regulatory processes such as authorisation procedures or risk minimisation activities. If their outcomes are adopted, their long‐term impact may be hardly perceptible or diluted amongst a set of other factors influencing regulatory practice. The example of quantitative methods for benefit–risk assessment illustrates the situation where outcomes are not considered mature enough by regulators to be implemented, or vice versa where the regulatory environment is not mature enough to adopt them. In such cases, their impact may still be measured by adoption in other research projects as a leg‐up for further advances in method development ultimately leading to regulatory improvement. Such contribution will have limited visibility in the final outcome. Finally, regulatory science may provide new facts and observations that contribute to better assess healthcare delivery, such as results of direct‐to‐patient research. Such knowledge may be implemented only through regulatory guidance but may also need to be quickly updated due rapid technological progresses. In conclusion, regulatory science is a scientific discipline embracing a large variety of activities and outputs, from new techniques and products to methodological standards and guidance. What unites them is the common objective to impact significantly on regulatory practice, medicines development or public health. Apart from tools and standards that may be immediately available, such impact may not be easily visible or directly measurable. An evaluation of how well the objective has been achieved should therefore be part of the planning of all regulatory science projects.

## Conflict of Interest

The authors declare no conflict of interest.

**Key Points**

- Regulatory science projects should aim to impact on regulatory practice, medicine development or public health, but such outcomes may not be visible or directly measurable. An evaluation of how well the objectives have been achieved should be part of the planning of all projects.
- Results of the PROTECT project had a significant impact on the performance of signal detection from EudraVigilance and approaches for multicentre database studies. Assessment and testing of methods for benefit–risk integration and representation had a major contribution for further developments in this field.

### Disclaimer

The author is a full‐time employee of the European Medicines Agency; the views expressed in this article may not be understood or quoted as being made on behalf of the European Medicines Agency or one of its Committees or working parties. The PROTECT project received support from the Innovative Medicine Initiative Joint Undertaking (www.imi.europa.eu) under Grant Agreement no. 115004, resources of which are composed of financial contribution from the European Union's Seventh Framework Programme (FP7/2007–2013), and the European Federation of Pharmaceutical Industries and Associations companies' in kind contribution.
